# Ankle and knee extensor muscle effort during locomotion in young and older athletes: Implications for understanding age-related locomotor decline

**DOI:** 10.1038/s41598-020-59676-y

**Published:** 2020-02-18

**Authors:** Juha-Pekka Kulmala, Marko T. Korhonen, Luca Ruggiero, Sami Kuitunen, Harri Suominen, Ari Heinonen, Aki Mikkola, Janne Avela

**Affiliations:** 1 Motion Analysis Laboratory, New Children’s Hospital, University of Helsinki and Helsinki University Hospital, Helsinki, Finland; 2 Faculty of Sport and Health Sciences, University of Jyväskylä, Jyväskylä, Finland; 3 School of Health and Exercise Sciences, University of British Columbia, Kelowna, BC Canada; 4 KIHU - Research Institute for Olympic Sports, Jyväskylä, Finland; 5 Department of Mechanical Engineering, Lappeenranta University of Technology, Lappeenranta, Finland; 6 Aspire Academy, Doha, Qatar

**Keywords:** Computational biophysics, Muscle

## Abstract

Age-related reduction in muscle force generation capacity is similarly evident across different lower limb muscle groups, yet decline in locomotor performance with age has been shown to depend primarily on reduced ankle extensor muscle function. To better understand why ageing has the largest detrimental effect on ankle joint function during locomotion, we examined maximal ankle and knee extensor force development during a two-leg hopping test in older and young men, and used these forces as a reference to calculate relative operating efforts for the knee and ankle extensors as participants walked, ran and sprinted. We found that, across locomotion modes in both age groups, ankle extensors operated at a greater relative effort compared to knee extensors; however, slightly less pronounced differences between ankle and knee extensor muscle efforts were present among older men, mainly due to a reduction in the ankle extensor force generation during locomotion modes. We consider these findings as evidence that reduced ankle push-off function in older age is driven by a tendency to keep ankle extensor effort during locomotion lower than it would otherwise be, which, in turn, may be an important self-optimisation strategy to prevent locomotor-induced fatigue of ankle extensor muscles.

## Introduction

Slowly but surely, ageing compromises locomotor ability by adversely affecting the performance of our muscles. However, although age-related force deficit has been shown to occur similarly across all major locomotor muscle groups^1,2^, decline in locomotor ability with age depends primarily on compromised ankle extensor muscle function. This is evident from numerous studies showing that older adults compared to younger adults walk and run with reduced ankle extensor moment and power generation while exhibiting little or no declines in the muscular output of more proximal lower limb muscle groups^3–8^. Although this so-called distal-to-proximal shift in joint kinetics during locomotion has been well-recognised for over two decades^4,7^, it remains unclear why, specifically, the function of the ankle joint becomes primarily affected by the ageing process.

A potential explanation for the above question is that, during walking and running, ankle extensors have to operate at a greater proportion of the maximal capacity compared with other lower limb muscle groups, and thus, they demonstrate a lower force reserve to buffer the age-related loss of muscle capacity. Previous studies in older adults examining relative muscle efforts during walking have relied on the normalisation procedure, where joint moments produced during gait are related to the maximal joint moments generated by the same muscle group during the dynamometer measurement^9–11^. However, this procedure has led to mixed findings regarding the question of whether older adults operate at a greater effort in their ankle extensors compared to other lower-limb muscle groups during walking. For example, while studies of Beijesberg *et al*.^10^ and Brown *et al*.^9^ suggest that, during walking, old adults’ ankle extensors work near their capacity limits and at almost twice the greater relative level compared to knee or hip extensors; however, the recent work of Spinoso *et al*.^11^ reported a much higher effort for the knee extensors (>90%) compared to ankle extensors (71%) during habitual walking.

Furthermore, although the normalisation of walking-related joint moments to dynamometer-derived maximal joint moments logically demonstrates a greater relative muscle effort for the old versus the young adults; the obtained efforts have been unrealistically high, often far exceeding the 100% reference level for maximum capacity even during habitual walking^9–12^. As a consequence, an analysis of relative muscle efforts in running would be subject to even larger uncertainty because peak joint moments are typically two to three times greater compared to those of walking^13^.

Unrealistically high relative effort values may have been the result of a different joint moment definition methods between gait (inverse dynamics approach) versus maximum reference force measurements (dynamometer method)^14^. To some extent, the normalisation procedure may also be influenced by the mismatch in muscle functional characteristics between locomotion (dynamic stretch–shortening type of muscle action) and the maximal dynamometer test (pure isolated muscle action)^15^. An additional and more direct method for determining muscle effort is to use the same inverse dynamics method in the locomotor task and the force reference test.

To our knowledge, Hortobágyi *et al*.^16^ were the first to study the knee extensor muscle efforts in selected daily activities utilising the identical inverse dynamics–based maximum force test. By relating joint moments during stair walking and rising from a chair task to the maximal moments obtained from an isometric leg press test, the researchers reported a reasonable approximation of knee extensor muscle effort across the studied activities for young and old adults (42–54% vs 78–88% of maximal capacity). Recently, by using the ‘matched method approach’ and the all-out hopping test as a reference for maximal muscle capacity, we reported fairly realistic knee and ankle extensor muscle efforts for level walking (19% and 35%), running (63% and 84%) and sprinting (72% and 96%) in young adults^17^. When comparing the results of young adults between our work and Hortobágyi *et al*. (2003), it can be noted that our knee extensor effort for level walking (19%) is 2.2 and 2.8 times lower, respectively, to knee extensor effort during stair descent (42%) and ascent (54%) in the study of Hortobágyi *et al*. (2003), which seems reasonable, as the knee joint moments are approximately 1.5–2.5 times lower during level walking versus stair walking^18,19^. To date, however, no studies have assessed the knee and ankle extensor muscle efforts during level locomotion in older adults using the inverse dynamics-based reference force test.

Therefore, to better understand why ageing has the largest detrimental effect on the ankle joint function during level locomotion, we measured maximal ankle and knee extensor forces during a two-leg hopping test in older and young men and used these as a reference to define relative efforts for the knee and ankle extensors as participants walked, ran and sprinted. Master athletes were recruited to serve as older group to ensure that all participants were able to successfully perform the two-leg hopping task. Based on our previous work in young men^17^, we hypothesised that relative muscle effort for all three modes of locomotion would be higher at the ankle versus the knee extensors in both age groups in the present study. In comparison to young men, we predicted that older men would demonstrate similar maximum force declines in the knee and ankle extensors during the hopping reference test when compared with young men; however, due to the age-related reduction of ankle kinetics in locomotion^3,4,6^, we expected that locomotor-related muscle efforts would show a less prominent increase at the ankle versus the knee extensors in older men compared with the young men.

## Results

### Knee and ankle extensor forces during walking, running and sprinting

**Figure 1:**
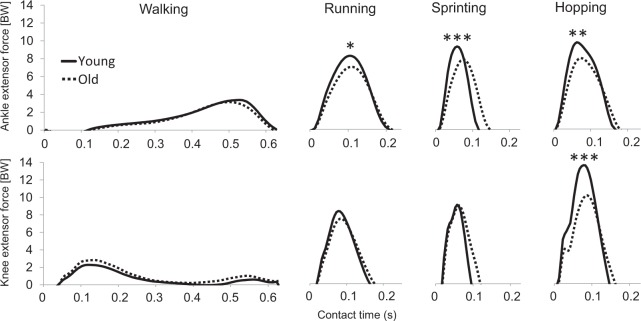
The knee and ankle extensor muscle forces (BW, body weight) during the stance phase of walking, running, sprinting and hopping for the young and older groups. Statistical significance between young and older groups (independent *t*-test): *p < 0.05, **p < 0.01, ***p < 0.001.

We recruited 13 healthy older (mean age 67 years) and 13 young men (mean age 27 years) who had several years of training experience in track and field events (running & long jump). Biomechanical analysis was used to determine body segmental movements and the knee and ankle extensors forces across walking, running and sprinting (see Methods section for details). Compared to the young men, older men walked with a similar self-selected speed (1.6 m/s) and exhibited a tendency towards a distal-to-proximal shift in muscular contributions, evident as a 8% lower ankle extensor force (*p* = 0.13, ES = 0.61) but a 6% greater knee extensor force (*p* = 0.27, ES = 0.46) (Fig. 1, Table 1). During running (4.1 m/s) and maximum speed sprinting (young 9.3 m/s vs old 7.4 m/s), older men showed a 17% (*p* = 0.006, ES = 1.19) and 18% (*p* = 0.001, ES = 1.48) lower ankle extensor force, respectively, compared with the young men, while somewhat smaller reductions were noted in the knee extensor force when running (11%, *p = *0.17, ES = 0.55) and sprinting (8%, *p* = 0.37, ES = 0.49) (Fig. 1, Table 1).

**Table 1:** Comparisons of the knee and ankle extensor forces and efforts during walking, running and sprinting in young and old groups.

	Walking		Running		Sprinting		Hopping reference test	
	Young	Old	Young	Old	Young	Old	Young	Old
**Extensor muscle force (BW)**								
Knee	2.71 ± 0.52	2.95 ± 0.53	8.43 ± 1.48	7.50 ± 1.86	9.82 ± 2.67	8.99 ± 1.89	13.74 ± 2.89	10.19 ± 1.60***
Ankle	3.37 ± 0.30	3.18 ± 0.32	8.37 ± 1.30	6.91 ± 1.15**	9.48 ± 1.24	7.75 ± 1.10***	9.92 ± 1.38	7.79 ± 1.88**
**Relative effort (%)**								
Knee	20.5 ± 5.7	29.5 ± 7.0***	63.6 ± 16.7	74.6 ± 17.9	72.5 ± 18.3	89.1 ± 16.2*		
Ankle	34.6 ± 6.1	42.9 ± 11.4*	84.8 ± 11.3	91.4 ± 16.8	96.2 ± 11.0	102.5 ± 16.2		
**Joint angle at peak force (deg)**								
Knee	24.3 ± 5.2	30.5 ± 4.9**	44.2 ± 5.1	43.6 ± 6.4	45.4 ± 7.4	45.0 ± 5.2	58.6 ± 7.4	57.8 ± 8.0
Ankle	14.6 ± 3.8	15.7 ± 5.6	26.5 ± 4.6	25.5 ± 4.5	24.8 ± 5.4	25.6 ± 4.0	29.0 ± 4.5	26.0 ± 6.1
**Joint angular velocity at peak force (degs**^**−1**^**)**								
Knee	−11.7 ± 36.1	−3.1 ± 28.6	0.8 ± 55.0	−15.7 ± 105.9	−1.3 ± 63.3	−34.2 ± 85.4	4.4 ± 129.6	−0.3 ± 74.6
Ankle	42.6 ± 26.4	45.7 ± 34.6	21.7 ± 43.6	10.6 ± 73.4	−38.8 ± 62.0	11.7 ± 77.3	−44.1 ± 74.9	−16.0 ± 43.7

Data shown as mean ± s.d.; BW, body weight.

Statistical significance between young and old groups (independent *t*-test): *P < 0.05; **P < 0.01; ***P < 0.001.

Relative efforts were determined by normalizing extensor muscle forces across different modes of locomotion to maximum force quantified in two-leg hopping. Positive angular velocities indicate shortening of the muscle-tendon units at peak force while negative values represent lengthening of the muscle-tendon units at peak force.

### Maximum knee and ankle extensor muscle forces in a hopping test

To determine the maximum forces each participant could develop from their knee and ankle extensors during natural dynamic movement, repetitive two-leg hopping as high as possible was performed^17^. The hopping height of the older men was 13 cm lower than the younger men (20.2 cm and 33.3, *p* = 0.001, ES = 2.3). The total limb force production, determined as a peak vertical GRF during the contact phase^20^, was 0.83 body weight (BW) lower in older versus young men (*p* = 0.001, ES = 1.6, Table 2). Muscle force analysis showed that the older men produced 26% lower maximum knee extensor force (*p* < 0.001, ES = 1.5) and 21% lower maximum ankle extensor force (*p* = 0.003, ES = 1.3, Table 1).

**Table 2:** Locomotor speed, ground contact time, peak vertical GRF and hopping height for the young and old groups.

	Walking		Running		Sprinting		Hopping reference test	
	Young	Old	Young	Old	Young	Old	Young	Old
Speed (m/s)	1.6 ± 0.1	1.6 ± 0.1	4.1 ± 0.1	4.1 ± 0.1	9.3 ± 0.4	7.4 ± 1.0***		
Contact time (s)	0.63 ± 0.02	0.63 ± 0.02	0.21 ± 0.02	0.21 ± 0.02	0.12 ± 0.01	0.14 ± 0.01***	0.17 ± 0.02	0.19 ± 0.02
Peak vertical GRF (BW)	1.23 ± 0.06	1.27 ± 0.09	3.11 ± 0.31	2.85 ± 0.30*	3.30 ± 0.35	2.93 ± 0.32*	3.76 ± 0.51	2.93 ± 0.54***
Hopping height (cm)							33.3 ± 5.6	20.2 ± 5.8***

Data shown as mean ± s.d.; BW, body weight.

Statistical significance between young and old groups (independent *t*-test): *P < 0.05; ***P < 0.001.

### Knee and ankle extensor efforts during walking, running and sprinting

**Figure 2:**
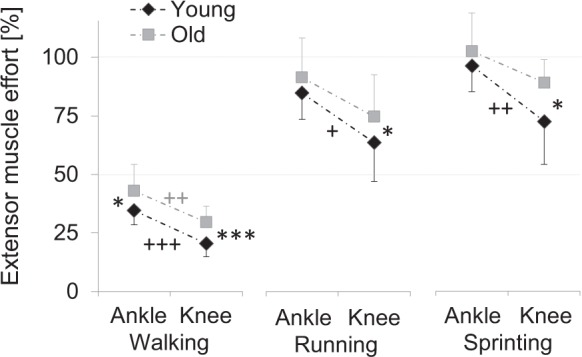
The knee and ankle extensor muscle efforts during the stance phase of walking, running and sprinting for the young and older groups Statistical significance between young and older groups (independent *t*-test): *p < 0.05 and ***p < 0.001. Within group difference (dependent *t*-test): ^+^*p* < 0.05, ^++^*p* < 0.01, ^+++^*p* < 0.001.

To estimate how hard the knee and ankle extensor muscles have to work while walking, running and sprinting, we related the forces of these muscles during locomotor tasks to maximum forces obtained from the hopping reference test. We found that ankle extensors generally worked at a greater proportion of their maximal capacity compared to the knee extensors (Fig. 2, Table 1). During walking, both young (*p* < 0.001, ES = 2.39) and old (*p* = 0.005, ES = 1.42) men had significantly greater operating efforts at the ankle extensors versus the knee extensors. However, during running (young: *p* = 0.009, ES = 1.49; old: *p* = 0.055, ES = 0.97) and sprinting (young: *p* = 0.005, ES = 1.57; old: *p* = 0.065, ES = 0.83), only young men exhibited ankle extensor effort values that were significantly greater than those of the knee extensors.

Finally, older men showed a general tendency towards greater operating efforts compared with young men (Fig. 2, Table 1). However, this was more pronounced at the knee extensor level across walking (knee extensors: *p* = 0.001, ES = 1.41; ankle: *p* = 0.029, ES = 0.91), running (knee extensors: *p* = 0.12, ES = 0.64; ankle extensors: *p* = 0.26, ES = 0.46) and sprinting (knee extensors: *p* = 0.022, ES = 0.96; ankle extensors: *p* = 0.26, ES = 0.45).

## Discussion

To better understand why the ankle joint specifically is a weak link in older adults’ locomotion^3–7^, we examined the ankle and knee extensor muscle efforts during walking, running and sprinting in older men and young men. Our results generally demonstrate that across locomotion modes ankle extensors in both age groups operate at greater effort compared to the knee extensors. However, the difference between ankle and knee extensor muscle effort was slightly less pronounced in older men, especially during running and sprinting; compared to the young men, this was mainly due to the greater tendency to reduce the ankle extensor force than the knee extensor force during locomotion.

Compared to ankle extensors, the knee extensors in older adults were capable of producing a more similar force during locomotion modes with the young men, although the maximum force development in our hopping reference test in older men showed virtually similar reduction at the knee (26%) versus the ankle extensors (21%). This suggests that a greater capacity reserve of the knee extensors in locomotion enabled older men to maintain the knee extensor force nearly similar to young men despite a substantial reduction in the maximum knee extensor force capacity. Therefore, unlike the ankle extensors, the knee extensors may not play a significant role in locomotor decline with age.

We interpret the present findings as evidence that biomechanical gait adaptations in older age are triggered by a tendency to keep ankle extensor effort during locomotion lower than it would otherwise be. Consequently, reduced ankle push-off may be an important strategy for older people to prevent locomotor-induced fatigue of the ankle extensors, which, in turn, can optimize the control and performance of prolonged locomotion.

On the other hand, the present findings further suggest that exercise interventions designed to slow down or even restore age-related ankle push-off deficit in locomotion should focus on improving ankle extensor muscle performance. Paradoxically, however, studies in older adults show no evidence that power training intervention^21^ or an active endurance running background^22^ could reverse distal-to-proximal shift in joint kinetics during walking. In fact, these studies^21,22^ have reported a tendency towards increased hip kinetics during walking in response to exercise, indicating a more pronounced distal-to-proximal shift with age. Consequently, these observations may indicate that ankle push-off deficit is an unavoidable part of natural ageing. However, more evidence is needed to confirm this hypothesis.

Importantly, when comparing the present results to previous studies, it can be noted that our muscle effort levels in walking are remarkably lower than those measured using dynamometer-derived maximum reference values^9–11^, while for running, a lack of representative studies prevents direct comparisons. The discrepancy in muscle effort levels during walking between the present and previous work^9–11^ arises from the fact that the maximum joint moments determined in our hopping test (See Supplementary Tables S1 and S2) are two to three times higher than those obtained from dynamometer measurements^9–11^. Given the methodological and functional aspects associated with these alternative approaches, we believe that the inverse dynamics–based reference force test can provide a more accurate representation of the true muscle efforts, as has also been suggested by others (e.g.^23^).

However, this study is not without limitations. First, because our analysis was limited to athletic participants with excellent physical condition, caution must be made in generalising these results to sedentary people with low functional capacities. It can be expected that such inactive persons would operate a much closer to their maximal force production capacities. Second, our inverse dynamics–based analysis was unable to account for the effects of muscle co-contractions and two-joint muscles when calculating the forces of the knee and ankle extensors, which may underestimate the true muscle forces generated during movements^24^. However, because all measured activities are similarly affected, it is unlikely that our primary findings are significantly influenced by this issue. Third, we cannot completely confirm that the two-leg hopping task enabled both extensor muscle groups to reach their maximum force in our participants. The final limitation is that we were unable to perfectly match the muscle contractile conditions (joint angles and angular velocities) between locomotor tasks and the reference force test. This can lead to the underestimation of muscle efforts particularly during walking, where the leg is more extended and the advantage from stretch-shortening cycle on muscle force production is less pronounced^25^. On the other hand, due to the relatively low angular velocities of both knee and ankle joints at the time of peak force (Table 1), it can be assumed that muscle contractile conditions remain nearly isometric and, thus, favorable for producing force across measured activities^26–29^. Consequently, an isometric joint angle-matched force test, similar to the study of Hortobágyi *et al*.^16^, could provide a reasonable reference for maximal muscle capacity. Such a reference test would also be easier to perform and, therefore, more suitable when attempting to quantify muscular efforts in persons with limited functional abilities.

## Methods

### Participants

We recruited 13 healthy older (age, 67 ± 9 years; height, 176 ± 7 cm; weight, 76 ± 12 kg) and 13 young (age, 27 ± 5 years; height, 183 ± 6 cm; weight, 76 ± 10 kg) men for this study. Each participant came from a background of competition (sprinters and long jumpers) and several years of training. The subjects provided informed consent and confirmed that they did not have a previous history of any musculoskeletal problems, such as a recent injury or surgery, which could have an effect on the movement patterns. The study was approved by the ethics committee of University of Jyväskylä, and performed in the accordance with the Declaration of Helsinki.

### Biomechanical analysis

Three-dimensional motion capture measurements were conducted in an indoor sports hall. First, after a thorough warm-up period, the participants performed three walking trials at a self-selected speed and three running trials at 4 m/s (±10%). Next, each participant twice sprinted 60 m at maximum effort. The locomotor speeds were measured using photocells positioned 30 m and 40 m along the track. The same part of track was used as a motion capture area. Finally, to determine the maximum forces each athlete could develop from their knee and ankle extensors during natural movement, measurement of a series of maximal two-legged hops were taken from each subject. Beginning with each foot standing on one of two force plates, each participant jumped repeatedly as high as possible over a period of 10 s, rested three minutes, and then repeated the 10 s jumping cycle. We selected this plyometric movement as a reference test, because it has been shown to enable humans to produce the greatest muscle moments from their knee and ankle extensors^30^.

For 3D motion analysis, anthropometric measurements (height, weight, leg length, and knee and ankle diameters) were taken for each subject, and 22 retro-reflective markers were placed bilaterally (on the shoe over the second metatarsal head and over the posterior calcaneus, lateral malleolus, lateral shank, lateral knee, lateral thigh, anterior superior iliac spine, posterior superior iliac spine, clavicula, sternum, seventh cervical vertebra, and tenth thoracic vertebra) on the subjects based on the Plug-in Gait full-body model (Vicon, Oxford, UK). An eight-camera system (Vicon T40, Oxford, UK) and five force platforms (total length 5.7 m, AMTI, Watertown, MA) were used to record marker positions and ground reaction force (GRF) data synchronously at 300 and 1500 Hz, respectively. To avoid impact artefacts, marker trajectories and GRF data were low-pass filtered using a fourth order Butterworth filter with cut-off frequency of 18 Hz^31^. Foot contact and toe-off events were determined based on the 20 N vertical GRF threshold level and Plug-in Gait model (Nexus v. 1.7, Vicon, Oxford, UK) was then used to analyse joint angles and moments for measured activities. Participants used their own running shoes during walking and hopping and their own track shoes during running and sprinting.

To avoid muscle fatigue, only two maximal sprinting trials were collected per subject. Therefore, the leg that demonstrated a greater number of clear force plate contacts on any of the five force plates during two sprinting trials was selected for the analysis. The total number of analysed contacts per subject was five during walking and running, and two to four during sprinting. During hopping, the three best trials were selected for the analysis based on the magnitude of the peak vertical GRF. The mean value of selected trials was used for each biomechanical variable.

In order to estimate how close to their individual maximal capacities do the knee and ankle extensors work during locomotion modes, we used a biomechanical modelling to calculate muscle forces during measured activities, and then related peak forces taken from each mode of locomotion to maximum forces produced by the same muscle group during the two-leg hopping task. Muscle force calculations rather than joint moments were selected for the primary analysis of relative muscle efforts because the former takes into account the variation in the muscle’s moment arm that occur especially at the knee due to different joint angles between measured activities. Nevertheless, we also report relative effort results calculated using the joint moment method in the Supplementary Material (Table S2).

In the primary analysis of relative muscle efforts, the muscle forces were calculated following previously published models for the knee^32^ and ankle^33^ extensors. The models assume that all limb muscles cross only a single joint and that no muscle co-contractions occur. Input variables for both models included joint angle and net extensor moment (*M*). First, the knee extensor muscle effective moment arm (*L*_knee_) was calculated as a function of knee flexion angle using non-linear equation:

1 $${L}_{{\rm{knee}}}=8.0{{\rm{E}}}^{-5}{x}^{3}-0.013{x}^{2}+0.28x+0.046;\,{\rm{where}}\,x={\rm{knee}}\,{\rm{angle}}$$

Second, knee extensor force (*F*_knee_) was calculated as follows:

2 $${F}_{{\rm{knee}}}={M}_{{\rm{knee}}}/{L}_{{\rm{knee}}}$$

Finally, ankle extensor force (*F*_ankle_) was determined by dividing the net ankle extensor moment by the estimated ankle extensor muscle lever arm (*L*_ankle_) as described by Self and Paine^33^:

3 $${F}_{{\rm{ankle}}}={M}_{{\rm{ankle}}}/{L}_{{\rm{ankle}}}$$

4 $${L}_{{\rm{ankle}}}=-\,0.5910+0.08297a\mbox{--}0.0002606\,{a}^{2};\,{\rm{where}}\,a={\rm{ankle}}\,{\rm{angle}}$$

### Statistical analysis

Statistical tests were performed with IBM SPSS software (Version 23.0, Chicago, IL, USA). Shapiro-Wilk and Levene’s tests were used to confirm the normal distribution and the equality of variances, respectively. We used a two-tailed dependent sample t-test to examine whether relative muscle efforts differed between the knee and ankle extensors within age groups. In addition, we used a two-tailed independent sample t-test to test whether locomotor speed, ground contact times, peak vertical GRFs, hopping height, muscle forces, muscle efforts, joint angles and angular velocities differed between young and older groups. *P* < 0.05 was considered significant. Symbols are used to describe statistically significant differences as follows: *P < 0.05; **P < 0.01, ***P < 0.001.

## Supplementary information

Supplementary Tables

## Data Availability

The datasets generated and/or analysed during the current study are available from the corresponding author on reasonable request.
