# Supplementary material for: Ankle and knee extensor muscle effort during locomotion in young and older athletes: Implications for understanding age-related locomotor decline
Source: Sci Rep. 2020 Feb 18;10:2801. doi: 10.1038/s41598-020-59676-y (PMC7028745; doi:10.1038/s41598-020-59676-y)
Supplement: Supplementary file 1 — Supplementary Tables [file 41598_2020_59676_MOESM1_ESM.docx]

Supplementary Materials for

**Ankle and knee extensor muscle effort during locomotion in young and older athletes: Implications for understanding age-related locomotor decline**

Juha-Pekka Kulmala^1^, Marko T Korhonen^2^, Luca Ruggiero^3^, Sami Kuitunen^4,5^, Harri Suominen^2^, Ari Heinonen^2^ Aki Mikkola^6^ & Janne Avela^2^

^1^Motion Analysis Laboratory, New Children’s Hospital, University of Helsinki and Helsinki University Hospital, Helsinki, Finland. ^2^Faculty of Sport and Health Sciences, University of Jyväskylä, Jyväskylä, Finland. ^3^School of Health and Exercise Sciences, University of British Columbia, Kelowna, BC, Canada. ^4^KIHU - Research Institute for Olympic Sports, Jyväskylä, Finland. ^5^Aspire Academy, Doha, Qatar. ^6^Department of Mechanical Engineering, Lappeenranta University of Technology, Lappeenranta, Finland.

Corresponding author: Dr. Juha-Pekka Kulmala [juhapekka.kulmala@gmail.com](mailto:juhapekka.kulmala@gmail.com)

This PDF file includes:

• Table S1.

• Table S2.

Table S1 summarizes the dynamometer-derived maximal joint moments measured in the previous studies examining muscular efforts during level walking and Table S2 presents joint moment data across movement tasks in the present study. Of note is, that the dynamometer-derived maximal knee and ankle joint moments (Table S1) are remarkably lower than those measured during hopping reference test (Table S2). Especially at the ankle joint level the dynamometer-derived maximal joint moment can be lower than joint moment produced during walking, suggesting that it may be particularly difficult to obtain comparable ankle joint moments from dynamometer test versus inverse dynamics analysis.

Table S2 further presents the relative muscular efforts calculated by using joint moments rather than muscle forces. The ankle extensor muscle efforts calculated via joint moment (Table S2) versus muscle force (Table 1) method are essentially similar, whereas the knee extensor muscle effort becomes somewhat greater when using the joint moment method. This results from the fact that the joint moment method does not consider muscle moment arm changes that occur with different joint angles. For example, the knee extensor muscle moment arm decreases as a function of increased knee flexion angle (Mason et al. 2008), and therefore, greater extensor muscle force is needed to produce the same joint moment if the knee joint is held in a more flexed position. Because the joint angles differ between locomotor tasks and the reference force test, we believe that the muscle force method may therefore provide more accurate estimates for muscle efforts in the present study.

**Table S1**. The magnitude of maximal extensor joint moments from relevant studies examining the knee and ankle extensor efforts during level walking.

**Table S2**. The peak extensor joint moments and muscle efforts (determined by the joint moment method) in young and old men in the present study.

Statistical significance between young and old groups (independent *t*-test): **P* < 0.05; ****P* < 0.001.

**References**

Brown, S. J. *et al.* Do patients with diabetic neuropathy use a higher proportion of their maximum strength when walking? *J. Biomech.* **47,** 3639–3644 (2014).

Spinoso, D. H. *et al.* Hip, Knee, and Ankle Functional Demand During Habitual and Fast-Pace Walking in Younger and Older Women. *J. Aging Phys. Act.* **27,** 242–251 (2018).

Samuel, D., Rowe, P. & Nicol, A. The functional demand ( FD ) placed on the knee and hip of older adults during everyday activities. *Arch. Gerontol. Geriatr.* **57,** 192–197 (2013).

Mason JJ, Leszko F, Johnson T, Komistek RD. Patellofemoral joint forces. J Biomech. **41**, 2337–48 (2008).
